# Minimally invasive surgical treatment of minimally displaced acetabular fractures does not improve pain, mobility or quality of life compared to conservative treatment: a matched-pair analysis of 50 patients

**DOI:** 10.1186/s13018-020-01611-y

**Published:** 2020-03-23

**Authors:** Benedict Swartman, Johanna Pelzer, Sven Yves Vetter, Nils Beisemann, Marc Schnetzke, Holger Keil, Paul Alfred Gruetzner, Jochen Franke

**Affiliations:** Medizinische Fakultät Heidelberg, Im Neuenheimer Feld 672, 69120 Heidelberg, Germany

**Keywords:** Acetabular fracture, Minimally invasive technique, Conservative therapy, Long-term outcome, Risk factors

## Abstract

**Background:**

Currently available procedures for the treatment of minimally displaced acetabular fractures include conservative treatment and minimally invasive percutaneous screw fixation. Screw fixation of acetabular fractures allows patients’ early full-weight bearing due to improved biomechanic stability. Can the range of motion, pain and mobility and quality of life in patients with acetabular fractures be improved by minimally invasive screw fixation, compared to conservative treatment in the long term?

**Methods:**

Patients treated for a minimally displaced acetabular fracture, either conservatively or by closed reduction percutaneous screw fixation, in the period from 2001 to 2013 were included in this retrospective study. Minimal displacement was considered to be less than 5 mm. As well as the collection and analysis of baseline data, Harris Hip Score, Merle d’Aubigné score and Short Form 12 (SF-12) questionnaire data were recorded in the context of a clinical study. To better account for confounding factors, patients of each group were matched. The matched-pair criteria included age, BMI, Letournel fracture classification and the presence of associated injuries.

**Results:**

Twenty-five patients from each group were matched. On the Harris Hip Score, conservatively treated patients obtained 96 points (52–100, SD 17) vs. 89 points (45–100, SD 17, *p* = 0.624). On the Merle d’Aubigné score, conservatively treated patients obtained 17 points (10–18, SD 2) vs. 17 points (11–18, SD 2, *p* = 0.342).

Patients with acetabular fractures treated by minimally invasive screw fixation did not result in improved quality of life, measured by SF-12 questionnaire, compared to conservatively treated patients (PCS 47, SD 9 vs. 44, SD 10; *p* = 0.294 and MCS 51, SD 7 vs. 53, SD 7; *p* = 0.795).

**Conclusions:**

The clinical results of the two groups revealed no statistically significant differences. From the data, it cannot be deduced that minimally invasive surgical therapy is superior to conservative treatment of minimally displaced acetabular fractures. Prospective randomised studies are recommended to allow reliable evaluation of both treatment options.

**Trial registration:**

Retrospectively registered

## Background

With an incidence of 3/100,000 per year, pelvic fractures account for about 2–8% of all fractures [1]. In 15.4% of cases, they are associated with acetabular fractures [2]. Isolated acetabular fractures account for 0.3–6% of all human fractures annually [2]. The uniformly advocated gold standard in displaced acetabular fractures according to the literature is open reduction and internal fixation [1, 3–6]. Anatomical reduction provides the best functional results in surgical management [7, 8].

In minor displaced fractures, the minimally invasive surgical approach is gaining increasing significance. Associated problems are the anatomically related narrow corridors for screw placement and the accompanying risk of complications [3, 5, 9]. Developments in the area of intraoperative navigation in particular are intended to reduce complications and to make minimally invasive techniques safer [10–13]. The percutaneous approach has already been studied with respect to reduction outcome and clinical outcome after 30 months, and the results were good to very good [14]. Conservative treatment appears to be at least a therapeutic option with satisfactory clinical results in particular in the area of minimally displaced acetabular fractures [15, 16].

To date, there has been no direct comparison of conservative and minimally invasive surgical therapy with respect to long-term clinical outcomes of minor displaced acetabular fractures. The hypothesis of this study is that minimally invasive surgical treatment of minimally displaced acetabular fractures results in a better clinical outcome than conservative therapy. Relevant parameters are the range of motion of the hip joint, pain and mobility (screened by Harris Hip score and Merle d’Aubigné score) and quality of life (screened by the Short Form 12 (SF-12) questionnaire).

## Methods

All acetabular fractures in a Level 1 trauma centre were recorded during the period 2001–2013. Patients with acetabular fractures treated by minimally invasive surgery or conservatively were included and studied.

In all operative cases, surgery was performed in percutaneous technique and in supine position. Screws were inserted solely by stab incision and either guided by conventional fluoroscopy or navigated (Brainlab, Munich, Germany). Reduction was achieved by different techniques such as manipulating the leg, placing instruments through stab incisions or merely by screw insertion and using its lag ability. Reduction and implant placement were verified with intraoperative imaging, in most cases by 3D imaging. Aftercare was the same in both groups: The patients were restricted to non-weight bearing on the injured side for 6 weeks. After that and after radiologic examination, they started weight bearing with 20 kg, increasing by 10 kg per week.

For the purposes of homogenisation and better comparability of the groups, a matched-pair analysis was performed. The pairs were matched on the basis of the criteria of age, BMI, Letournel classification and associated injuries. Value ranges were defined within these criteria, which the partner likewise had to meet. An overview of the defined ranges is provided in Table 1. Letournel classification parameters were split into two halves, which represent elementary and associated fractures (Letournel 1–5 and 6–10).

**Table 1:** Matched-pair criteria

Age (years)	<30	30–39	40–49	50–59	60–69	70–79	≥ 80
BMI (kg/m^2^)	<18.5	18.5–24.9	25–29.9	≥ 30			
Letournel	1–5	6–10					
Associated injuries	Yes	No					

Classification of ranges for pairing in the matched-pair analysis. The partners in a pair were within the same range of values in all categories

Patients, which were converted into total hip arthroplasty within the follow-up period, were excluded for a second analysis to gain data with less functional bias.

Patients were identified retrospectively and then followed-up for the scores. Harris Hip Score, Merle d’Aubigné score and the SF-12 questionnaire (PCS and MCS; Physical and Mental Health Composite Scores) were recorded. A minimum follow-up period of 15 months was presumed.

Data were collected and documented using Excel 2010 (Microsoft, Seattle, USA) and statistical analysis was performed using Excel 2010 and SPSS (SPSS Inc., Chicago, USA). Student’s *t* test for normally distributed variables and the Mann-Whitney *U* test for non-normally distributed variables were used to compare the groups. Ordinal scales were investigated by the Wilcoxon signed-rank test. The level of significance was defined as *p* < 0.05.

## Results

During the period 2001–2013, 689 patients with acetabular fractures were treated. Of these, 241 were managed conservatively, 383 by open surgery and 47 by minimally invasive surgery. Twenty-five patients from the minimally invasive group could be recruited for the follow-up study and paired on the basis of the previously described matching parameters with 25 patients from the conservative group. Consequently, 50 patients were included in the study (45 men and 5 women). The median age at the time of the accident was 56 years (19–80; SD 15). The over-all mean follow-up period was 5.8 years (1–13; SD 5.6). The mean follow-up period in the conservatively treated group (group 1) was 7.8 years (1–13; SD 6.8) and in the surgically treated group (group 2) 3.7 years (1–10; SD 2.8). These values differed significantly (*p* = 0.009).

**Fig. 1:**
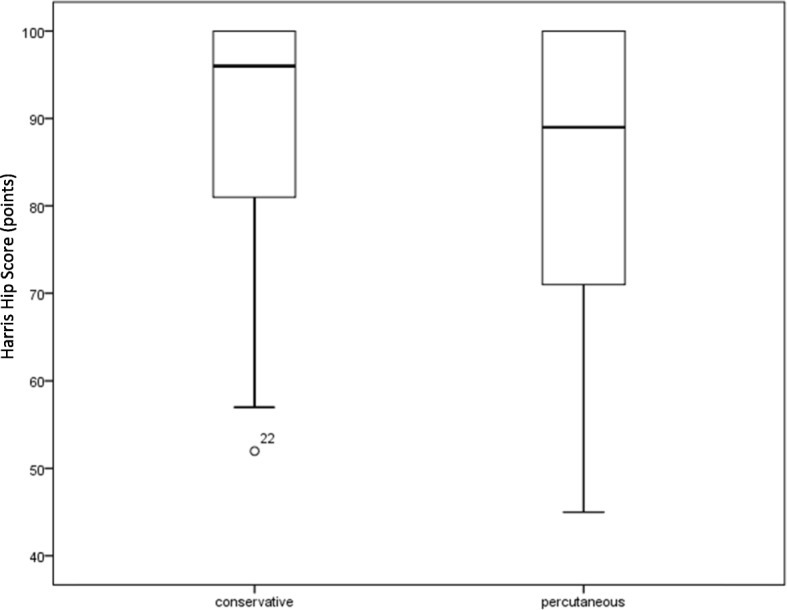
Harris Hip Score. The Harris Hip scores of the two treatment groups following treatment of acetabular fractures do not differ significantly. The groups contain 25 patients each

**Fig. 2:**
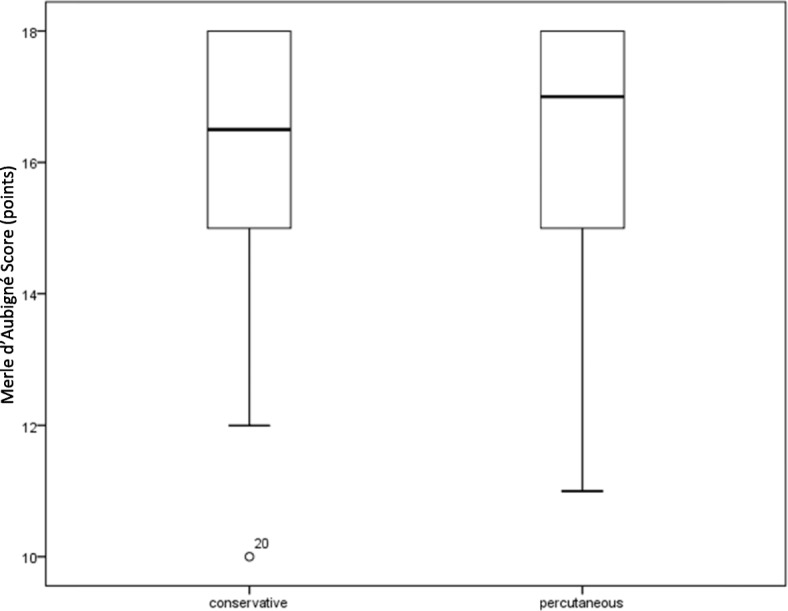
Merle d’Aubigné score. The Merle d’Aubigné scores of the two treatment groups following treatment of acetabular fractures do not differ significantly. The groups contain 25 patients each

Conservatively treated patients (group 1) obtained 96 points (52–100; SD 17) on the Harris Hip Score, whereas the operated group (group 2) obtained 89 points (45–100; SD 17; *p* = 0.624; Fig. 1). On the Merle d’Aubigné score, conservatively treated patients (group 1) obtained 17 points (10–18; SD 2), while the operated patients (group 2) obtained 17 points (11–18; SD 2; *p* = 0.342, Fig. 2).

Three patients were converted into total hip replacement during the follow-up period in each group. Repeated statistical analysis without these patients showed similar results: On the Harris Hip Score, group 1 obtained 96 points (SD 17; 52–100) and group 2 obtained 93 points (SD 18; 45–100) without a significant difference between the groups (*p* = 0.749). On the Merle d’Aubigné score, group 1 obtained 17 points (SD 2; 10–18) and group 2 obtained 17 (SD 2; 11–18) points. A significant difference was not found (*p* = 0.516).

**Fig. 3:**
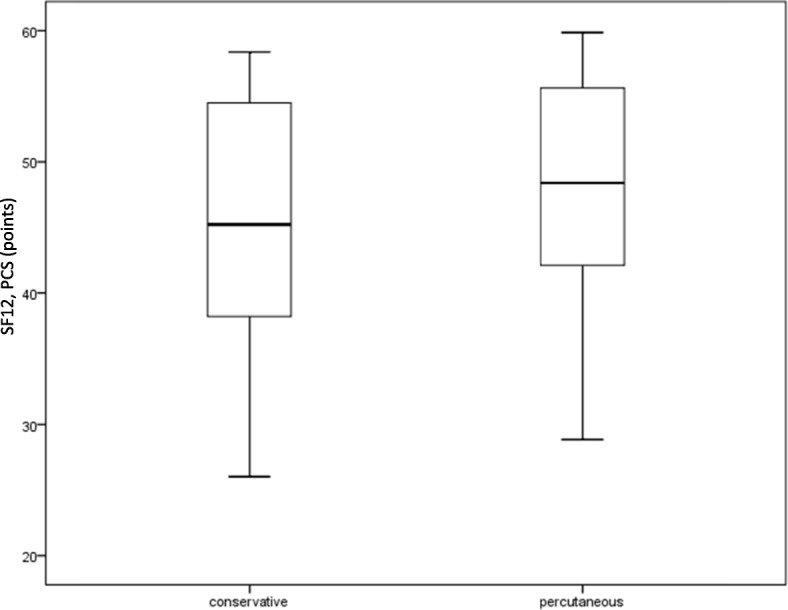
SF-12, PCS. The PSC (Physical Health Composite Score) of the SF-12 of the two treatment groups following treatment of acetabular fractures do not differ significantly. The groups contain 25 patients each

**Fig. 4:**
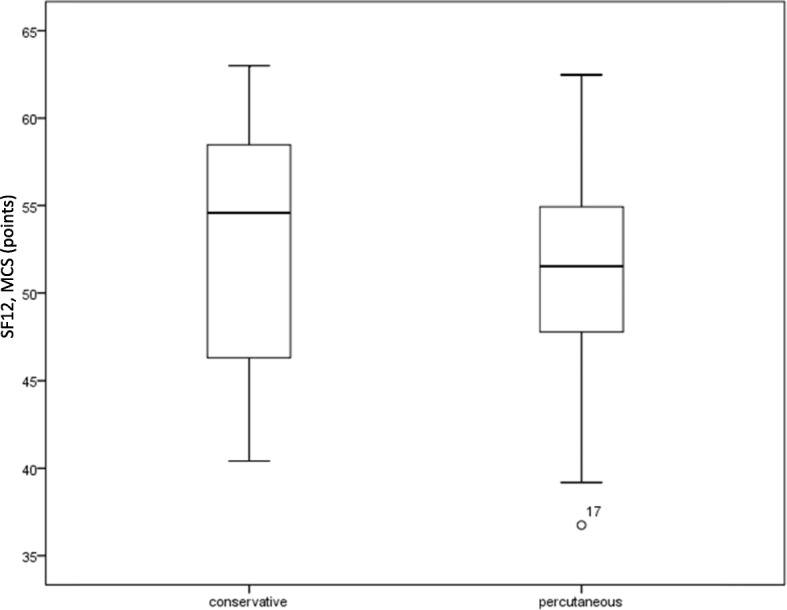
SF-12, MCS. The MSC (Mental Health Composite Score) of the SF-12 for the two treatment groups following treatment of acetabular fractures do not differ significantly. The groups contain 25 patients each

On the SF-12 PCS, group 1 obtained 44 points (SD 10; 25–58) and group 2 obtained 47 points (SD 9; 28–59) without significant difference between the groups (*p* = 0.294, Fig. 3). On the SF-12 MCS, group 1 obtained 53 points (SD 7; 40–64) and group 2 obtained 51 points (SD 7; 39–63). There was no significant difference with *p* = 0.795 (Fig. 4).

## Discussion

The aim of this study was to compare the conservative treatment approach to acetabular fractures with the minimally invasive surgical approach in a matched-pair analysis. This study sought to investigate whether the minimally invasive surgical procedure was superior in terms of long-term range of motion, pain, mobility and quality of life. Following a mean follow-up period of 5.78 years, no significant difference was observed between conservatively treated and minimally invasive surgically treated acetabular fractures on the Harris Hip, Merle d’Aubigné and SF-12 outcome scores.

Open reduction and internal fixation are undoubtedly regarded as the gold standard in displaced acetabular fractures [1, 3–6]. It has been shown to achieve comparable functionality and gait pattern in the injured and the non-injured limb [4, 17]. There is no standard definition as to when a fracture may be considered “displaced”. Grubor et al. describe a degree of dislocation of more than 5 mm as the threshold for opting for surgical treatment [15]. They considered this threshold when indicating operative treatment in 85% of patients (*n* = 96). There was no difference in clinical outcome scored by the Harris Hip Score after 18 months between the operative and conservative group. Magala et al. stated a fragment dislocation of 2–3 mm in the acetabular roof and 4–5 mm elsewhere in the acetabulum leading to necessary operative treatment [16]. A recommendation for surgical management that is solely dependent on the localisation of the fracture in the main load-bearing area of the acetabular roof has been proposed by some authors [18, 19].

According to biomechanical studies, screw fixation of anterior column posterior hemi-transverse (ACPHT) acetabular fractures shows the same stability as standard buttress plate fixation [20]. Under full-body weight load, fractures of the posterior wall resulted in dislocations of only < 1 mm [21]. Percutaneous screw fixation exhibits a higher resistance than plate fixation in the management of transverse acetabular fractures [22]. It can therefore be assumed that early mobilisation is obtained following minimally invasive screw fixation of acetabular fractures.

The Merle d’Aubigné score has yet to be validated as an outcome parameter of acetabular fracture. Nevertheless, it is the most commonly used tool for ascertaining clinical outcome following this injury [23, 24]. Moed et al. studied 150 patients with acetabular fractures after a mean follow-up of 5 years (range 2–17) in order to validate the Merle d’Aubigné score in relation to the Musculoskeletal Function Score [24]. The patients all underwent operative treatment of Acetabular fractures. The authors found a strong relationship between the Musculoskeletal Function Assessment and the Merle d’Aubigné score. They described a mean Merle d’Aubigné score of 16.8 points (range 9–18); this value is consistent with the result of our population of minimally invasive surgically treated patients with 16.44 points (range 11–18) and approximates to that of our conservatively treated population of 15.82 points (range 12–18).

Schwabe et al. reported very good clinical results after closed reduction and 3D-fluoroscopy navigated percutaneous screw fixation in acetabular fractures after 30 months [14]. On the Harris Hip Score, the patients obtained 92.4 ± 6.8 points. There was no control group in terms of conservative treatment. Further investigations comparing both treatment options were performed by Grubor et al. [15]. Grubor et al. reported good and very good results in the Harris Hip Score after a follow-up of 18 months in 56% (open surgically treated) and 57% (conservatively treated), respectively. Pairs had not been matched.

In our study, the results of the Harris Hip Score were lower than reported by Schwabe et al., although conservative and minimally invasive surgical treatment showed almost equal results. The follow-up period of 5.78 years is considered a long-term follow-up. Since Grubor et al. investigated open surgical procedures, their report does not answer the question about the superiority of minimally invasive over conservative treatment.

Neither Briffa et al. [1], Dodd et al. [23], Grubor et al. [15] or Schwabe et al. [14] have investigated the quality of life in their reports on clinical outcomes after acetabular fractures. Since the SF-12 form seems to be the most sensitive tool in order to detect reduced pre-traumatic quality of life in patients with acetabular fractures [25], it may also be the most reliable parameter in order to investigate the postoperative quality of life in the long term. Borg et al. reported a quality of life with the SF-36 lower than the norm in patients undergoing open surgery after acetabular fractures after a follow-up of two years [26].

In our opinion, quality of life is a central aspect of clinical outcome after extremity or pelvic injuries. Therefore, we evaluated the quality of life with the SF-12 form and found no significant difference between minimally invasive and conservatively treated patients after acetabular fractures.

One limitation of the study arises from the different follow-up periods of the two treatment groups. The follow-up study of the conservative group occurred significantly later than that of the minimally invasive surgery group. The reason for this lies in the gradual establishment of surgical and also navigation-assisted procedures in our facility, which are increasingly replacing the conservative approach in the management of acetabular fractures. We assume a 15-month period as sufficient in order to complete the healing process after acetabular fractures. Thus, a comparison of the functional outcome measures seems appropriate. However, secondary complications need to be critically scrutinised in this context, such as the number of total hip replacements performed by the time of the follow-up study. We only investigated the long-term outcome and cannot give any information on short-term follow-up. Possibly, early differences are masked but irrelevant in the long term.

Letournel classification parameters were split into two groups for matching the pairs. Dividing the classification into Letournel 1–5 and 6–10 seemed feasible, since it differentiates between elementary and associated fractures [27]. Nevertheless, it has to be taken into account that under a clinical point of view, a posterior wall fracture (type 1) and an anterior column fracture (type 4) do not have more in common than a posterior wall fracture (type 1) and a posterior wall/posterior column fracture (type 6). Looking at the distribution, we found most of the patients within group 1–5 were classified Letournel 4, whereas in the other group (6–10) most of them were categorised under Letournel 9 and 10. This seems to provide a relevant difference, suitable for matching pairs.

We tend to recommend conservative treatment of acetabular fractures where open surgery is not urgently required. We are unable to define a threshold value of the degree of dislocation in favour of the minimally invasive or conservative approach as an aid to decision-making. Nevertheless, it is our view that a treatment decision must always be taken individually on the basis of the patient’s attendant circumstances. The assumption of stability to full-weight bearing following minimally invasive surgical management of acetabular fracture should be included in the decision-making process.

In our study, the indication for treatment was not established on any absolute degree of dislocation. The creation of comparator groups was based simply on the treatment option used. The Letournel classification was not recorded with the aim of including the degree of injury as a criterion for grouping but rather for ensuring that in the matching process the classification was homogeneously distributed in both groups. In our opinion, this produces better comparability and ultimately a higher predictive value of the study. Since not all conservatively treated patients had CT scans taken, the exact fracture dislocation prior to treatment could not be evaluated. There was no questionnaire or radiologic assessment of preliminary arthritis of the hip joints. Yet, we considered comparable groups due to the matching procedure as mentioned above.

## Conclusion

A superiority of minimally invasive surgical treatment over conservative therapy of acetabular fractures in the clinical outcome could not be observed. Where open reduction with internal fixation is not necessary due to the degree of dislocation, the possibility of conservative therapy should therefore always be considered. We also recommend an individual treatment decision based on the patient’s attendant circumstances.

## Data Availability

The datasets used and/or analysed during the current study are available from the corresponding author on reasonable request.

## Abbreviations

BMI: Body mass index
CT: Computer tomography
MCS: Mental Health Composite Score
PCS: Physical Health Composite Score
SD: Standard deviation
SF-12: Short Form 12
SPSS: Superior performing software system
